# Medical practitioners’ confidence in performing paediatric critical procedures in the emergency department

**DOI:** 10.4102/jcmsa.v3i1.209

**Published:** 2025-09-19

**Authors:** Shivanthra Ramdass, Matthew Zoghby, Nicholas Dufourq

**Affiliations:** 1 Department of Emergency Medicine, Faculty of Medicine, University of KwaZulu-Natal, Durban, South Africa; 2 Interdepartmental Division of Critical Care, Faculty of Adult Critical Care, University of Toronto, Toronto, Canada

**Keywords:** paediatric critical procedures, perceived confidence, emergency department, paediatric emergencies, continued medical education

## Abstract

**Background:**

Paediatric patients make up a significant portion of emergency department (ED) visits, and critically ill children require timely, life-saving procedures. While medical practitioners (MPs) may have the necessary skills, a lack of confidence can prevent them from applying these skills effectively, potentially impacting patient outcomes. This study aimed to assess MPs’ confidence in performing critical procedures for paediatric patients and to identify factors influencing it.

**Methods:**

This is a cross-sectional descriptive study, conducted in four EDs in KwaZulu-Natal between 07 July 2023 and 11 November 2023. A questionnaire completed by MPs with confidence levels ranked on a five point Likert Scale was used.

**Results:**

With a 68% response rate the mean level of confidence was 3 [s.d. = 12.5]. Emergency medicine specialists when compared to non-specialists had a higher level of confidence being 3.99 (*P* = 0.02). The amount of time spent treating paediatric patients did not affect confidence levels. Performing a procedure more frequently generally resulted in a higher level of confidence apart from that of newborn cardiopulmonary resuscitation and cardioversion.

**Conclusion:**

The confidence of MPs (translating to 60% confident) was significantly less than their perceived importance of the procedure. This could suggest motivation to improve their ability to perform these skills. The rank of doctor and frequency of procedures performed can affect an MP’s confidence. Medical practitioners being confident when performing critical procedures in paediatric patients can possibly improve outcomes, which needs to be researched further.

**Contribution:**

MPs may require ongoing practical medical training or simulations to boost their confidence during paediatric procedures.

## Introduction

Medical practitioners (MPs) working in an emergency department (ED) should be able to confidently perform critical and life-saving procedures required to treat a range of conditions in children; whether they are confident doing so is not always known.^1^ The paediatric population makes up a significant population group treated in mixed EDs ranging from 13% in South Australia to 18% in Sweden with the age range being from 0 to 18 years.^2^ During 2023–2024, the Australian Institute of Health and Welfare reported 19.9% of ED visits were children below the age of 14 years.^3^ In 2020, Mitchells Plain Hospital in South Africa treated 9982 paediatric patients out of a total of 39 905 patients that presented to the ED from 27 February 2020 to 04 June 2020, making up 25% of all ED presentations.^4^ In 2019, Cabalatungan et al. stated that 85% of the paediatric population in the United States of America were treated in general EDs as opposed to paediatric EDs.^1^

Paediatric patients presenting to an ED are considered a special population because of their unique needs.^5^ In 2012, paediatric patients in South Africa were defined as patients below the age of 14 years; however, other countries included patients up to the age of 18 years.^6,7^ These patients have anatomical and physiological differences when compared to adults.^8^ They have differing degrees of psychological maturity and have unique social and emotional needs when compared to adults.^5^ In paediatrics, the spectrum of diseases is markedly different from adults.^5^ They may initially mask the severity of their illness, thereafter deteriorating rapidly; the history is often obtainable only from their caretakers; the examination is complicated by lack of co-operation; vital signs are tricky to measure and normal values change with age; equipment needs to be adapted to the size of the child and drug doses must be calculated by weight.^5,9^ Despite these challenges, paediatric patients who are critically ill require timely and effective life-saving interventions to ensure optimal outcomes.^10^ Those trained in emergency medicine should be able to perform critical procedures required to treat a range of conditions in children, which include complex paediatric resuscitations.^11^

In a study by Simon et al., emergency physicians expressed significant discomfort in the handling of many life-saving paediatric procedures.^12^ In addition, a study by Chen et al. demonstrated that just over half of the MPs in the study population felt confident in managing acutely ill children.^10^ The reasons for lack of confidence in MPs are multifactorial, including the emotional response to the critically ill child, the lack of perceived competence in technical abilities required and the maintenance of critical procedures in paediatric patients.^12,13^ This highlights potential training gaps in procedural paediatric emergency medicine, particularly in settings where such procedures are frequently encountered.^12^ In noting the lack of confidence in managing acutely ill children, which includes procedural paediatric emergency medicine, one needs to consider why confidence is important. Gottlieb et al. in a conceptual review found that confidence and competence are linked without confidence being a synonym for competence.^14^ It is further explained that both confidence and competence need to be aligned to have the optimal outcome without problems, which is referred to as manifest competence.^14^ This skill set is extremely important, as found by Craig et al. in a survey assessment done in 2016, where 90% of respondents thought it extremely important to maintain the skills of performing critical procedures in paediatric patients in the ED.^11^ They also believed that an MP’s confidence in performing these skills should be consistently supported and maintained.^11^

As a lower to middle class income country, South Africa bears a heavy burden of paediatric disease, with under 5 mortality rate in 2012 was recorded at 40 per 1000 live births.^15^ It was further found that KwaZulu-Natal (KZN) had the highest mortality rates in 2012.^15^ Precise figures for ED visits in South Africa are not available; however, a study in the Western Cape found that 25% to 30% of ED patients were paediatric patients.^4,9^ Data regarding MPs’ confidence in performing critical procedures for paediatric patients in South African EDs are limited, as existing studies have assessed a combination of adult and paediatric critical procedures.^16,17^ Furthermore, some studies that were conducted did not include the doctors in ED.^18,19^

By determining whether MPs working in EDs in South Africa are confident in performing paediatric critical procedures, one can determine whether additional training is needed. Implementing such training may help to provide effective paediatric emergency care in EDs.

The objective of this study was to determine the confidence of MPs in performing critical procedures for paediatric patients in mixed EDs. An additional objective was to determine what factors affect the perceived level of confidence in performing critical procedures in paediatric patients.

## Research methods and design

### Study design

This was a cross-sectional descriptive quantitative study.

### Setting

The study took place in established EDs in KZN. These EDs belong to referral hospitals and receive paediatric patients referred for trauma, burns, general surgery pathologies, fractures and snake bites.

### Study population and sampling strategy

The study population included MPs working in the described departments. Qualified independent MPs comprising medical officers, Emergency Medicine Registrars and Emergency Medicine Specialists were included. Community service officers and interns were excluded from the study. This study took place between 07 July 2023 and 11 November 2023. The sampling strategy used was a combination of purposive and convenience sampling. To reduce social bias from the limited sampling period, department heads were informed about the study from the outset when granting permission. The heads of each department were asked to identify a day when all the staff would be present, allowing researchers to approach them for the study. On the scheduled day, they asked all department members to be present for data collection and to learn about the study in order to decide whether to participate. On this day, the questionnaire was presented to all staff members present at the time. Those who consented to participate were given the opportunity to take part.

### Data collection

The perceived level of confidence of MPs in performing critical procedures on paediatric patients was investigated using a questionnaire (Online Appendix 1) with a Likert Scale (ranging from having no confidence to being extremely confident). The questionnaire was adapted from a questionnaire used in a similar study after gaining consent from the author of that study.^11^ The questionnaire that was received from the referenced study was modified and adapted by removing questions that were not pertaining to the research questions in this study as well as by refining the list of critical paediatric procedures that were looked at. This questionnaire was not piloted. The definition of self-reported confidence was explained to them as a group when approaching each department with a pre-scripted introductory speech. It was defined as their own individual assessment of their ability to perform the stated tasks and not on the outcome of those tasks. The questionnaire also included the MPs’ perceived importance of the critical procedures and possible factors that may influence their level of confidence when performing these procedures. All data were collected on days when the departments met for their departmental meetings. The study was explained to the department as a group, and questionnaires were then distributed within the department. Those individuals who consented to participating completed a questionnaire and dropped it inside a sealed box placed outside the meeting venue. This box was then collected by the author at the end of the departmental meeting. The questionnaire did not have the respondent’s name, and so the consent forms were collected separately. All data were then handled solely by the author, entered and stored on a Microsoft Excel spreadsheet (Microsoft Office 2016, Microsoft Corporation) on a password-protected computer.

### Data analysis

Descriptive statistics were used to summarise the data. The study data were exported into IBM SPSS version 28 for statistical analysis. Descriptive statistics showed the participants’ demographic and professional profiles as well as details on their practices in the critical procedures. The descriptive statistics were reported in frequencies and percentages for categorical variables and as means with standard deviation (s.d.) for continuous variables. To explore the possible relationships, comparisons were made using Chi-square Statistical tests for categorical data and *t*-test or the Wilcoxon Rank-Sum test for numerical data. Levels of confidence in performing the critical procedures were compared according to the MPs’ demographics and profiles using one-way analysis of variance (ANOVA). The same method was used to determine if the level of confidence in performing a procedure was associated with the last time that procedure was performed. The Pearson correlation coefficient was used to examine if there existed a linear relationship between the level of confidence in performing the procedures and the perceived importance of performing those procedures. Statistical significance testing was set at the 95% confidence level and thus, *P* < 0.05 denoted statistical significance.

### Ethical considerations

Ethical approval was granted by a Biostatistics Research Ethics Committee in KwaZulu-Natal on 28 July 2023 (Protocol reference number: BREC/00005328/2023) and the Provincial Department of Health (National Health Research Database [NHRD] reference: KZ_202306_014). A formal detailed consent form was signed by each participant. The data were collected and recorded only by the author of the study to maintain participant confidentiality.

## Results

A total of 50 MPs participated in the study, representing 68% of the total number of MPs (*N* = 74) were approached. Medical practitioners who were absent during the questionnaire distribution within the respective departments did not take part in the process. There were missing responses for two questions in two questionnaires and, based on this, the data were accordingly adjusted and analysed. According to a five-point Likert Scale, the mean overall confidence across all procedures included in the study was 3 (s.d. = 12.5), while the mean perceived overall importance was 4.4 (s.d. = 9.4). Participants expressed the highest mean level of confidence when performing procedural sedation in paediatric patients (4 out of 5). The lowest mean level of confidence was expressed when inserting umbilical vein lines (2.5 out of 5). There was a higher level of confidence when performing cardiopulmonary resuscitation (CPR) for a child (mean confidence level = 3.4) compared to a newborn (mean confidence level = 2.6). The breakdown of their level of confidence in all other procedures is displayed in Table 1.

**TABLE 1a:** Critical procedures with the related mean level of confidence

Procedure	Mean level of confidence	s.d.
Overall	3.0	12.5
Procedural sedation	4.0	1.1
Child CPR	3.4	1.2
ETT insertion	3.3	1.4
IOL insertion	3.2	1.3
Laryngeal mask airway	3.2	1.3
Newborn CPR	3.0	1.3
Defibrillation	2.9	1.4
Tube thoracostomy	2.9	1.4
Central line insertion	2.7	1.5
Cardioversion- electrical/ chemical	2.6	1.4
UVL insertion	2.5	1.3

UVL, umbilical vein line; IOL, intraosseous line; ETT, endotracheal tube; CPR, cardiopulmonary resuscitation; s.d., standard deviation.

**TABLE 1b:** Critical procedures with the related mean level of confidence and mean level of confidence comparisons.

Mean level of confidence comparisons	Comparisons				
	Comparison subdivisions	*n*	Mean level of confidence	s.d.	*P*-value
Compared to percentage of hours spent treating paediatric patients	-	-	-	-	0.52
	0–25% of hours	37	3.10	1.19	-
	25–50% of hours	13	2.86	1.00	-
Gender comparison	-	-	-	-	0.07
	Male	20	3.39	0.98	-
	Female	30	2.8	1.19	-
Level of training comparison	-	-	-	-	0.02
	Consultant	9	3.99	1.20	-
	Registrar	5	3.11	1.06	-
	Medical Officer	36	2.79	1.03	-

UVL, umbilical vein line; IOL, intraosseous line; ETT, endotracheal tube; CPR, cardiopulmonary resuscitation; s.d., standard deviation.

The majority of the participants’ patient load was not paediatrics as described in Table 1. The number of hours spent treating paediatrics showed no statistically significant effect on the MPs’ level of confidence in performing these procedures.

Despite there being fewer male participants than female participants as shown in Table 1, they had a higher level of confidence overall (3.39 vs. 2.8), which was statistically insignificant (*P* = 0.07). Most participants attended more than one emergency medicine course (Table 2). The courses that showed the most impact on an MP’s level of confidence were Paediatric Advanced Life Support (PALS) (attended by most of the participants: *N* = 37) and Emergency Triage Assessment and Treatment (ETAT). Basic Life Support (BLS) had the least influence on an MP’s level of confidence. The course with the second lowest effect on an MP’s level of confidence was Advanced Trauma Life Support (ATLS). Consultants had an overall greater level of confidence compared to the other ranks of participants (*P* = 0.02) as shown in Table 1. This was followed by registrars and then the medical officers. Thus, the higher the rank of the MP, the greater the mean level of confidence.

**TABLE 2:** Summary of the courses attended by the participants.

Courses attended	*P*	Mean confidence level, if attended	Mean confidence level, if did not attend	*P*-value of the difference of attendance
BLS	36	3.04	3.01	0.94
PALS	37	3.12	2.77	0.34
APLS	8	3.25	3.00	0.57
ETAT	10	3.39	2.95	0.28
ATLS	35	3.06	2.98	0.82

BLS, basic life support; PALS, paediatric advanced life support; APLS, advanced paediatric life support; ETAT, emergency triage assessment and treatment; ATLS, advanced trauma life support.

**FIGURE 1:**
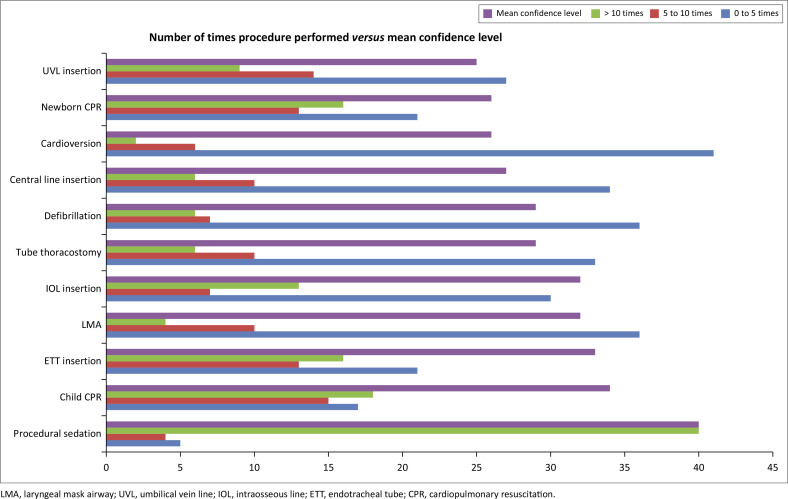
Comparing the number of times a procedure was performed to the overall mean level of confidence. The x-axis has two units: (1) The mean confidence level is the Likert value × 10; (2) The rest is the number of participants that performed the procedures at those frequencies.

Figure 1 looks at the number of times a procedure was performed in an MP’s career compared to the level of confidence. It illustrates that an increased frequency of performing certain procedures results in a greater overall mean level of confidence. This relationship is statistically significant with a *P*-value of < 0.05 for all procedures except cardioversion with a *P*-value of 0.08 and newborn CPR with a *P*-value of 0.26.

**FIGURE 2:**
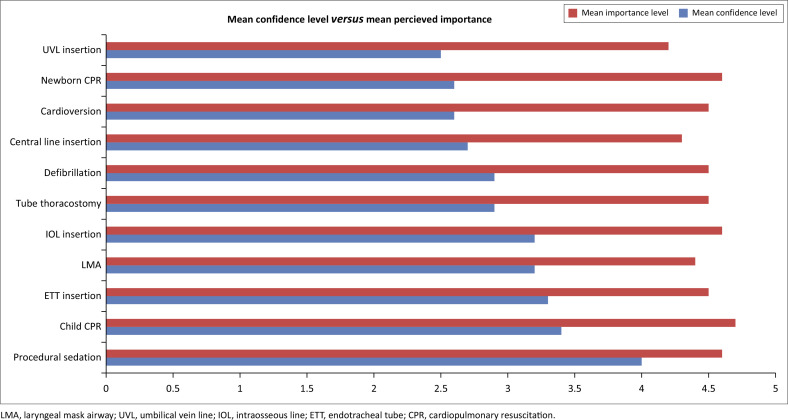
Comparison of the mean level of confidence and mean level of perceived importance (Mean Likert values).

Figure 2 shows that the MPs’ mean level of confidence differed significantly from their perceived importance of the procedures with a *P* < 0.001 for all procedures, except procedural sedation with a *P* < 0.003. Cardiopulmonary resuscitation of a child was regarded as the most important procedure followed by CPR of a newborn.

## Discussion

This study evaluated the self-assessed confidence of MPs performing critical paediatric procedures in various KZN EDs as well as the contributory factors. Results indicated a mean confidence level of 3 out of 5 on the Likert Scale translating to 60% across all procedures. Higher rank and greater procedural experience were associated with increased confidence.

Regarding the mean overall confidence level, similar results were obtained by Dufourq et al. demonstrating a self-assessed competence when performing paediatric emergency skills of 2.9 on a Likert Scale of 5.^17^ It is important to note that this study looked at self-assessed competence and not self-assessed confidence.^17^ These are two related but distinctly separate concepts.^21^ However, ED MPs were not included and the critical paediatric skills assessed were limited.^17^ Goldman et al. studied the confidence of MPs in caring for paediatric patients in EDs and looked at MPs’ perceptions of care for children in Rhode Island community EDs.^22^ Comparable results to this study were found with most participants reporting comfort in caring for acutely ill and injured children, with a median Likert score of 3.^22^ However, this comparison reflects an association only, because caring for children is not the same as performing critical procedures in children, although it may involve such procedures.^22^ Chen et al. studied that when looking at emergency physicians’ and residents’ perspectives on paediatric emergency medicine training programmes in Taiwan, they also looked at their confidence in managing paediatric patients.^10^ The results showed that only 52.3% felt confident in managing acute paediatric visits.^10^ Again, this comparison is one of association as managing acute paediatric visits may entail performing critical procedures for them.^10^ Therefore, comparable literature is sparse, and there is no established value for an adequate level of confidence when performing critical procedures in paediatrics. Despite this, it is important to consider that a parent may want someone who is extremely confident to perform critical procedures for their acutely ill child, as high confidence may contribute to better decision-making and quicker responses in emergency situations. This was considered by Gottlieb et al., who suggested that confidence and competence need to align.^14^ Self-confidence assists people in carrying out actions as well as completing them efficiently.^14^ Kim et al. have shown a moderate association between confidence and the knowledge of a skill.^23^ In light of the aforementioned information, it is recommended that strategies to enhance MPs’ confidence in performing paediatric critical procedures be carefully considered for implementation.

### Factors affecting confidence levels

Rank plays a crucial role in perceived confidence. The statistically significant relationship between rank and the mean level of confidence demonstrated that as the rank increased, the mean level of confidence increased. This could imply that the further training that is received to move up in ranks assists in confidence. However, Connick et al. showed a lower level of procedural confidence for consultants compared to specialist registrars stating that this may be because of registrars needing to perform more procedures as a training requirement.^24^ In addition, the years of experience and seniority in each rank have not been considered in this study. As there are conflicting results, further research is required to assess the true impact of rank on self-assessed confidence in performing procedures.

Confidence in performing procedures increased with the frequency of performance as shown in Figure 1. This suggests that repeated practice leads to higher self-perceived confidence. This resonated with a study done by Connick et al., showing that the more frequently a procedure was performed, the greater the practitioner’s confidence.^24^ Although studies conducted outside of South Africa indicated that these procedures were performed infrequently, a study in South Africa found that 71% of respondents had participated in resuscitations in paediatric patients over a 2-year period.^25^ The study indicates a higher frequency of critical procedures performed in South Africa; however, it also identified notable gaps in resuscitation management in paediatric patients, which may involve critical procedures.^25^ Present research showed that most of the participants performed all procedures apart from procedural sedation, zero to five times in their career. These findings indicate that additional practice may be necessary to improve confidence levels for most critical procedures involving paediatric patients.

In this study, most participants attended more than one emergency course. Paediatric Advanced Life Support and ETAT demonstrated a statistically significant improvement in mean confidence levels; however, these courses did not encompass all of the procedures evaluated in this study. The procedures not covered by PALS are laryngeal mask airway insertion, central line insertion, umbilical vein line insertion and procedural sedation. However, there was no comparable relationship between the confidence level of people who attended PALS and the procedures covered by PALS. The course with the lowest mean level of confidence was ATLS. This is most likely because the course focuses on trauma management in adults. This suggests that the current paediatric courses alone are not enough to maintain one’s confidence levels in performing all critical procedures in paediatric patients.

The study showed that gender may affect confidence levels with men being more confident than women with a *P*-value of 0.07 (mean level of confidence of men was 3.39 compared to women’s being only 2.8). This is not statistically significant. However, a similar trend was found by Selman et al. when evaluating plastic surgery residents’ operating abilities.^26^ Madrazo et al. found that women tend to underestimate themselves more than men in self-assessment scores.^27^ This is possibly because of a sex-based bias, where women unconsciously assume that they will perform poorly because of a stereotypical assumption that men are better than women.^26^ Although Dufourq et al. in their study did not show a statistically significant difference between genders, Connick et al. showed greater confidence in men compared to women.^17,24^ Therefore, gender does not have a statistically significant impact on mean confidence levels when undertaking critical procedures in paediatric patients.

### Importance of paediatric clinical procedures

The skills necessary to conduct critical procedures in children are considered extremely important.^11,28^ This was echoed in present research showing the overall importance of performing critical procedures in paediatric patients in the ED was 4.35 on the Likert Scale. Comparably, in 2018, Craig et al. did a multi-centre cross-sectional survey of senior emergency medicine MPs working in EDs, and even though the critical procedures were not performed frequently, they were still considered important.^11^ In this study, most participants were paediatric specialists; therefore, the perceived need for maintaining the skill set for critical procedures in children may have been biased.^11^ Mittiga et al. reported that over 90% of the 262 participants considered maintaining the studied set of paediatric critical procedures to be highly important.^28^ However, 68% of these institutions had paediatric emergency fellowship programmes, suggesting greater exposure to paediatric emergency care that may have influenced physicians’ views on maintaining critical procedural skills in children.^28^ When an MP believes a procedure is important to result in positive patient outcomes, they will be motivated to engage in performing that procedure.^25^ Vogt et al. showed that general practitioners are motivated to maintain and perform a procedure if they believe that it is effective and important.^29^ These studies, together with the present research, highlight the extreme importance of efficiently performing critical procedures in paediatric patients. The present research shows a large discrepancy between the mean level of confidence and the perceived importance of the studied critical procedures. The fact that the study showed a large discrepancy when correlating confidence and importance possibly indicates that MPs believe that the procedures are effective and are motivated to receive continued medical education for these procedures.

This study has thus allowed for important considerations. To get the best outcomes for paediatric patients in the ED, one needs to consider improving MPs’ confidence in performing critical procedures in paediatric patients.^14,23,26^ It is recommended to increase the practice of these procedures and to pursue ongoing medical education related to them, regardless of participation in standard emergency courses. Previous studies have shown that reinforcement improves confidence and commitment to change.^23^ Craig et al. in their study suggested the framework of learn, see, practise, do and maintain to confidently perform procedures.^11^ This includes ‘rapid cycle deliberate practice’, which involves repeated supervised attempts at resuscitation procedures with specific feedback and coaching.^11^ Further research is required to observe if this study’s results are reproducible for the rest of South Africa. This includes research regarding the best method in our setting to improve confidence in performing these procedures. However, this study does highlight the need to report back to the participating EDs suggesting further practical training to improve their MPs’ confidence in performing these skills.

### Limitations

The limitation of this study is that, firstly, it is a small study which includes MPs only from predetermined EDs in KZN. Secondly, the majority of these EDs are general EDs and only treat limited paediatric emergencies. The exposure to the number of paediatric patients in these EDs is limited; this may affect the perceived confidence. The lack of an extended sampling period may limit generalisability, as well as the questionnaire, despite being adapted from a prior study, was not piloted. The number of years of experience of the participants was not taken into consideration, and the last time they took part in the training courses mentioned was also not considered, which may affect the results of the study. Finally, this study looks at self-perceived confidence when an MP performs this skill set, which is subjective and may suggest bias.

## Conclusion

Despite the limited exposure to paediatric patients in KZN mixed EDs, paediatric critical procedures are perceived to be extremely important, and MPs are not completely confident when performing these procedures. The findings indicate that practice and training enhance confidence, whereas emergency courses alone are insufficient to bolster confidence in this skill set. The results suggest that MPs are motivated to further develop this skill set. Additionally, the study underscores the importance of exploring the relationship between confidence and competence. Sharing these insights with participating hospitals may encourage ongoing medical training in performing critical procedures in paediatric patients, potentially incorporating simulation-based training.
